# Psychosocial Health and Physical Activity in People With Major Depression in the Context of COVID-19

**DOI:** 10.3389/fspor.2021.685117

**Published:** 2021-10-29

**Authors:** Robyn Cody, Jan-Niklas Kreppke, Johannes Beck, Lars Donath, Anne Eckert, Christian Imboden, Martin Hatzinger, Edith Holsboer-Trachsler, Undine E. Lang, Sebastian Ludyga, Sarah Mans, Thorsten Mikoteit, Anja Oswald, Anja Rogausch, Nina Schweinfurth, Lukas Zahner, Oliver Faude, Markus Gerber

**Affiliations:** ^1^Department for Sport, Exercise and Health, University of Basel, Basel, Switzerland; ^2^Psychiatric Clinic Sonnenhalde, Riehen, Switzerland; ^3^Department of Intervention Research in Exercise Training, German Sport University Cologne, Cologne, Germany; ^4^Adult Psychiatric Clinics (UPKE), University of Basel, Basel, Switzerland; ^5^Private Clinic Wyss, Münchenbuchsee, Switzerland; ^6^Psychiatric Services Solothurn, Solothurn, Switzerland

**Keywords:** psychosocial health, COVID-19, depression, physical activity, attitudes, lockdown

## Abstract

**Introduction:** Major depression is a psychiatric disease associated with physical inactivity, which in turn affects mental and physical health. A randomized controlled trial is being implemented to facilitate physical activity in people with major depression. In March 2020, Swiss state authorities temporarily legislated a lockdown to contain the Coronavirus disease-19 (COVID-19), which influenced health, behavior and research. The aim of this study was to find out whether data gathered before and during/after the lockdown among in-patients with major depression differ with regard to psychosocial health, physical activity and related attitudes and to establish whether baseline data have been affected by the lockdown.

**Methods:** This is a cross-sectional analysis within a randomized controlled trial. Physically inactive, adult in-patients diagnosed with major depression were recruited from four Swiss psychiatric clinics between January 2019 and December 2020. Psychosocial health was measured with questionnaires pertaining to stress, sleep and health-related quality of life. Physical activity was measured with the Simple Physical Activity Questionnaire. Explicit attitudes were measured with seven questionnaires pertaining to physical activity-related motivation and volition. Implicit attitudes toward physical activity were captured with a single target implicit association test.

**Results:** The sample consisted of 165 participants (*n* = 119 before lockdown, *n* = 46 during/after lockdown). No statistically significant differences were found between in-patients with major depression assessed before and during/after the COVID-19 lockdown with regard to psychosocial health (stress, *p* = 0.51; sleep, *p* = 0.70; physical component of health-related quality of life, *p* = 0.55; mental component of health-related quality of life, *p* = 0.64), self-reported physical activity (*p* = 0.16) and explicit as well as implicit attitudes toward physical activity (*p* = 0.94). Hence, the COVID-19-induced lockdown seems not to have led to group differences.

**Conclusion:** Baseline data gathered in in-patients suffering from major depression who are physically inactive upon admission to in-patient treatment in Switzerland seem to be unaffected by the COVID-19-induced lockdown. To assess changes in said population regarding psychosocial health and physical activity patterns over time, longitudinal data are needed.

## Introduction

Major depressive disorder (MDD) is a psychiatric disease that affects 10–15% of people worldwide at least once in their lifetime (Lépine and Briley, 2011). Twenty to thirty percent of cases are chronic, lasting 2 years or longer (Riso et al., 2002) engendering suffering for the afflicted person and their social network from a psychological, medical, economical as well as social point of view (Pincus and Pettit, 2001). Population studies have found that depression symptoms as well as major depression are associated with double the risk of premature mortality (SMR = 1.9 in men; SMR = 2.1 in women); significantly higher risk of all-cause mortality (HR = 1.05; 95% CI = 1.02–1.10); and double the risk of cardiovascular, specifically stroke, mortality (OR = 2.24; 95% CI = 1.37–3.60) compared to general populations (Lépine and Briley, 2011). The relationship between MDD and physical activity seems to be bi-directional (De Moor et al., 2006; Lindwall et al., 2014). Longitudinal data show that people suffering from MDD tend more toward sedentary behavior (Firth et al., 2016; Stubbs et al., 2018). Thus, physical activity recommendations according to the World Health Organization stating a minimum of 150 min of moderate-to-vigorous physical activity per week (WHO, 2010) may not be adhered to. According to a meta-analytic analysis including data from North America, Europe and Australia, people with MDD spend 126 min (95% CI = 91.90–160.10) and 8.5 hours (95% CI = 7.51–9.62) per day engaging in all types of physical activity and sedentary behavior, respectively. Compared to healthy people, they spend a mean difference of −11.6 min (95% CI = −25.70–2.60) less being physically active and a mean difference of −0.2 h (95% CI = −19.7–0.8) more in sedentary behavior. These differences are deemed statistically significant (*p* < 0.05; Schuch et al., 2017). On the other hand, low levels of physical activity may perpetuate symptoms and even increase the risk for MDD (Mammen and Faulkner, 2013). This is also supported by meta-analytic data, which states that compared to people engaging in low levels of physical activity, those engaging in high levels have lower odds to develop MDD (OR = 0.83; 95% CI = 0.79–0.88), and that physical activity can have a protective effect across ages and geographic regions (Schuch et al., 2018). Inactivity leads to negative outcomes such as obesity, diabetes, more severe negative mental health symptoms and poor socio-occupational functioning (Firth et al., 2016).

Reasons for inactivity among people suffering from psychiatric illnesses can be physical co-morbidities such as obesity and diabetes as well as demographic influences such as sex (female), age (inverse) and lower educational level (Gerber et al., 2016a; Schuch et al., 2017). Additionally, psychological determinants correlated with engaging in health behaviors such as motivation, self-efficacy and volition may be impacted in people with MDD (Bauman et al., 2012; Vancampfort et al., 2015; Cortis et al., 2017). In recent years, increasing evidence points toward behavior being a result of two different information processing systems; one deliberate system requiring reflection, the other automatic requiring minimal cognitive resources (Chaiken and Trope, 1999; Kahneman and Frederick, 2002; Strack and Deutsch, 2004). Hence, behavior can be considered deliberative and rational decision-making enhanced by automatic, subconscious processes (Calitri et al., 2009; Conroy et al., 2010; Hyde et al., 2010). Decision-making is a slow process running on self-regulatory resources such as planning and goal setting. These are explicit motivational processes regulating intentional physical activity (Deutsch and Strack, 2006; Vohs, 2006). Additionally, intrinsic motivation may increase positive attitudes toward physical activity, thus leading to greater participation in and adherence to a physically active lifestyle (Haase et al., 2010). Attitudes are not only regulated by explicit motivational processes but also by automatic evaluative processes (Strack and Deutsch, 2004). Implicit attitudes have been shown to explain behavior beyond explicit attitudes (Greenwald and Banaji, 1995; Calitri et al., 2009; Rebar et al., 2016). They can be described as mental associations between a concept (e.g., physical activity) and its positive or negative evaluation (Chen and Bargh, 1999; Calitri et al., 2009). Automatic evaluative processes may influence immediate decisions regarding the target behavior (Brand and Schweizer, 2015); however, changes within the system are slow and gradual (Deutsch and Strack, 2006). Non-conscious processes explain the repetitious nature of physical activity, aspects unaccounted for in intention and maintenance when self-control resources are depleted (Aarts et al., 1997; Dimmock and Banting, 2009; Friese et al., 2011; Marteau et al., 2012; Rebar et al., 2016). Thus, pre-existing positive automatic evaluation of exercise may act as a buffer for potentially negative experiences (Antoniewicz and Brand, 2016a). Furthermore, implicit attitudes have been shown to be associated with previous self-reported physical activity, attentional biases to physical activity cues (Calitri et al., 2009), and may predict automatic / unplanned physical activity (e.g., taking the stairs instead of the lift) as well as objectively measured physical activity (Conroy et al., 2010).

In December 2019, first cases of Coronavirus disease-19 (COVID-19) were reported in China (WHO, 2021). By February 2020, the first cases were confirmed in Switzerland and a state legislated lockdown commenced in March until the end of April 2020. During the months between April and October, many restrictions were lifted; shops and restaurants re-opened, school recommenced as usual and sport facilities were accessible again. However, with increasing number of cases, protective measures were implemented again in October 2020. This included wearing masks in all public areas, restrictions of gatherings and recommendations for working from home (BAG, 2020). This ongoing pandemic is impacting lives as well as research in a multitude of ways. People may be experiencing mental health issues and decreases in well-being and mood, which in turn may be associated with negative impacts on lifestyle behaviors (Ammar et al., 2020). In an Australian nation-wide online survey, Stanton et al. (2020) found that people reporting negative changes in physical activity behavior had higher depression, anxiety and stress symptoms [see also Lindwall et al. (2014), Gerber et al. (2020), Meyer et al. (2020)]. These negative changes may have occurred because of the inaccessibility of gyms and outdoor spaces. When comparing online questionnaires before and during lockdown completed by healthy individuals in France and Switzerland, an increase of moderate physical activity and walking of about 10 min per day was found, whereas a decrease in vigorous physical activity became evident. Additionally, an increase in sedentary behavior of about 75 min per day was observed. Increased physical activity showed positive effects on physical health, whereas increased sedentary behavior had a negative influence on physical and mental health (Cheval et al., 2020). In line with findings from Stanton et al. (2020), changes in health behaviors are attributed to practical reasons, such as fewer opportunities to be physically active (Cheval et al., 2020). In addition, affective factors such as anxiety and stress are highlighted since they negatively influence mood and interest in activity (Cheval et al., 2020). Bi-directional effects of decreased mental health and physical activity have also been observed. According to an online survey conducted in the United Kingdom, a lockdown-induced reduction in physical activity may lead to increased symptoms of loneliness and depression, hence negatively impacting mental health (Creese et al., 2020). Not only do decreased physical activity levels influence mental health, self-isolation measures as experienced during quarantine may evoke feelings of anxiety and depression. These have been linked to long quarantine durations, fear of infection, frustration, boredom, inadequate supplies and information, financial loss and stigma (Brooks et al., 2020). For ongoing clinical trials, the COVID-19 pandemic has led to trials being stopped or paused. Issues such as missing data, incomplete follow-up, reduced on-site data monitoring with implications for data quality and integrity, missed treatments, changes in usual care and heterogeneity of patients included in trials are becoming prevalent in trial work (Kunz et al., 2020; Sathian et al., 2020). An analysis of data gathered before the pandemic can be helpful for deciding how to proceed (Kunz et al., 2020). Potential confounding caused by COVID-19 may also be detected by examining outcome rates before and during the pandemic (Tuttle, 2020). Trials, which have continued have been subject to decreased recruitment rates because of the need to reduce face-to-face interactions, and alternatives such as video conferences to obtain informed consent as well as collect data are being explored. Communication with clinic staff and sponsors has increased to develop individual solutions regarding COVID-19 implications for access to patients and funding matters. Additionally, some interventions have been interrupted or adapted to adhere to social and physical distancing standards (Mitchell et al., 2020). This gap can be filled with tools developed in recent years such as telehealth and home-based testing or monitoring technologies. There have also been cases in which recruitment rates have recovered, where attention must be payed to the potential effect COVID-19 may have on the study outcomes. For example, pre-existing conditions may have changed and lifestyle changes may have been made to adhere to safety guidelines (Tuttle, 2020).

Given the fact that changes in physical activity, as well as psychosocial health, have been detected in COVID-19 associated research so far, a closer look at data collected during this time is warranted. Hence, the aim of this study was to assess any potential differences in psychosocial health (stress, sleep, health related quality of life), self-reported physical activity and explicit as well as implicit attitudes toward physical activity in in-patients suffering from MDD recruited and assessed before COVID-19 in Switzerland and those recruited and assessed during/after the COVID-19-induced lockdown. It is of interest to see if trends that have been recognized in the afore-mentioned cohort studies are also visible in this sample. Additionally, it is of importance for the conduct of this trial to assess whether the COVID-19-induced lockdown in March and April 2020 has impacted the integrity of the baseline data in light of the planned longitudinal analyses.

## Materials and Methods

### Study Design

This is a cross-sectional analysis conducted within the PACINPAT randomized controlled trial (Physical Activity Counseling in In-Patients with Major Depression) taking place in four centers in three Swiss cantons (Basel, Solothurn, and Bern) (Gerber et al., 2019). Recruitment started in January 2019, paused from March to April 2020 because of a COVID-19-induced lockdown and was taken up again in May 2020 at half the rate (January 2019–February 2020, *N* = 143, M = 10 participants per month vs. March 2020–December 2020, *N* = 52, M = 5 participants per month). Participants were screened by clinic personnel and included in the trial upon providing written informed consent to a member of the study team. An individual, in person baseline assessment was completed during the first weeks of in-patient treatment conducted by a member of the study team. This consisted of a 90 min session in which demographic data, information regarding past and current depressive episodes, medication, secondary diagnoses, self-reported physical activity, and self-perceived fitness were collected via interview. Additionally, self-perceived fitness was assessed with a 1-item question: “Overall, how would you rate your physical fitness?” This question was answered on a scale ranging from 1 (very poor fitness) to 10 (excellent fitness). Psychosocial health (psychosocial stress, sleep and health-related quality of life) and explicit attitudes (physical activity-related intention, self-concordance, self-efficacy, action and planning coping, outcome expectancies, perceived barriers, and social support) toward physical activity were assessed with corresponding questionnaires (see below for more details). Implicit attitudes toward physical activity were assessed with a computer-based single target implicit association test. At this time, the participants did not receive any instructions regarding physical activity from the study team.

### Participants and Procedures

The current study sample consists of in-patients who were being treated for unipolar major depressive disorder at the time of recruitment and data collection. The inclusion criteria were as follows: women and men between 18 and 65 years, ICD-10 diagnosed depressive episode (single episode or recurrent), Beck Depression Inventory score (BDI) of at least 17 representing clinical depression, physical inactivity as defined by <150 min of moderate-to-vigorous physical activity per week prior to in-patient treatment, and adequate German language skills. The eligible patients, who provided written informed consent were included in the study. The patients were recruited at varying durations of clinic stay, and were receiving various pharmacological and complementary treatments. Physical activity 1 week prior to admission to in-patient treatment was measured with the International Physical Activity Questionnaire (IPAQ) in which days per week and minutes per day spent in physical activity are used to calculate a total amount of minutes per week spent in moderate-to-vigorous physical activity. According to a validation and reliability study, this measure is sufficiently representative of physical activity behavior (Craig et al., 2003). Depression severity was measured with the BDI, a 21-item self-report instrument used to assess the symptoms and attitudes referring to depression. Items are rated from 0 to 3 in terms of intensity, individual item scores are added to reach a total score. Scores bellow 10 indicate no or minimal depression, scores between 10 and 18 mild-to-moderate depression, scores between 19 and 29 moderate-to-severe depression and scores between 30 and 63 severe depression (Beck et al., 1988). According to meta-analytic analysis, the BDI has high internal consistency, content validity, sensitivity to change, and validity in differentiating between subjects. Thus, its psychometric properties are acceptable and recognized world-wide (Richter et al., 1998). Six patients were recruited despite a BDI score <17 based on the lead physician's estimation of sufficient depression severity. In addition, depression severity was assessed using the Hamilton Depression (HAMD) scale, which is a structured clinical interview. Scores range from 0 to 52 with higher scores representing more severe depression symptoms. This is a valid instrument with high discriminatory sensitivity (Fava et al., 1982). In addition, comparative reliability and validity have been established (Endicott et al., 1981).

All procedures received ethical approval from the “Ethikkommission Nordwest- und Zentralschweiz” and the “Ethikkommission Bern” (approval number: 2018-00976). Additionally the PACINPAT trial is registered in the ISRCTN registry (ISRCTN10469580) and the study protocol is published for more details (Gerber et al., 2019). All procedures are in line with the guidelines for Good Clinical Practice (ICH GCP) and with the ethical standards defined in the Declaration of Helsinki.

### Measures

Internal consistency of the questionnaires for the current study was measured by calculating the Cronbach's alpha (including 95% CIs) for each questionnaire individually. Values of ≥ 0.70 were deemed as satisfactory (Bland and Altman, 1997).

#### Psychosocial Health

##### Psychological Stress

The Perceived Stress Scale (PSS) was used to assess the degree to which, in the past month, life was appraised as unpredictable, uncontrollable and overloaded (Cohen et al., 1983). The ten questions were answered on a scale from 1 (never) to 5 (very often) (e.g., “In the last month, how often have you been upset because of something that happened unexpectedly?”). To obtain the score, positive items (questions 4, 5, 7, and 8) were reversed, then the total sum over all items was calculated, hence the score ranges from 0 to 40, with higher scores reflecting higher perceived stress levels. A review on the psychometric properties revealed Cronbach's alpha ranging from 0.78 to 0.91 across cultures (Lee, 2012). A German translation of the tool was validated in a representative German sample with satisfactory internal consistency and construct validity (Klein et al., 2016). The internal consistency in the present sample was satisfactory (Cronbach's alpha: α = 0.80, 95% CI = 0.74–0.84). The mean score across both groups was M = 37.14 (95% CI = 36.28 to 37.99).

##### Sleep

To measure symptoms and consequences of insomnia, which can be a common complaint in MDD (WHO, 2004), the seven-item Insomnia Severity Index (ISI) covering sleep complaints over the last 10 weeks was used. Three items relate to onset and maintenance (during the night and in the morning) of sleep on a scale from 0 (no difficulties) to 4 (very difficult). One item addresses satisfaction with sleep pattern, also ranging from 0 (very satisfied) to 4 (very dissatisfied). The next item refers to the extent to which the sleep problem interferes with daily functioning ranging from 0 (not at all) to 4 (very much). A further item relates to the perceived noticeability of others regarding the link between sleep problems and impaired quality of life of the person in question also ranging from 0 (not at all) to 4 (very much). The last item captures the degree to which the sleep problem causes distress on a scale from 0 (not at all) to 4 (very much). The final score is computed by the sum of all items which ranges from 0 to 28 with higher scores indicating higher levels of insomnia (0–7 = no clinical insomnia, 8–14 = subthreshold insomnia, 15–21 = moderate clinical insomnia, 22–28 = severe clinical insomnia). This is a self-reported measure which can be completed in 5 min (Bastien et al., 2001). Reliability as well as concurrent and content validity were deemed satisfactory for the original English questionnaire (Bastien et al., 2001) as well as the German translation (Gerber et al., 2016b) used in this study. The internal consistency in the present sample was satisfactory (α = 0.77, 95% CI = 0.71–0.82). The mean score across both groups was M = 12.11 (95% CI = 11.24–12.98).

##### Health-Related Quality of Life

The Medical Outcomes Study Short Form 12 (SF-12) was used to assess health-related quality of life. This questionnaire originated in a longer form, SF-36 including eight domains: general health, physical functioning, social functioning, role limitations caused by physical problems, role limitations caused by emotional problems, mental health, vitality and bodily pain. The reliability of this questionnaire has been tested in both general and psychiatric populations, and has been proven to be internally consistent and valid in people with depression (McHorney et al., 1994; Leidy et al., 1998). It was found that two factors, the Physical Component Summary (PCS) and Mental Component Summary (MCS) account for >80% of the variance of individual scales with reliability estimates usually exceeding 0.90 (Ware et al., 1994). Additionally, these factors require fewer items and are easy to interpret. In the shorter form (SF-12) the PCS and MCS have been found to correlate highly with those of the SF-36 (Ware et al., 1996). Sound psychometric properties of the SF-12 in people with severe mental illness have been proven (Salyers et al., 2000). Both PCS and MCS scores range from 0 to 100 with higher scores indicating higher health-related quality of life correspondingly. In the present sample, the internal consistency was satisfactory for both the PCS and MCS (α = 0.76, 95% CI = 0.70–0.82 and 0.70, 95% CI = 0.62–0.77, respectively). The mean scores across both groups were M = 49.21 (95% CI = 47.83–50.60) for the Physical Component Summary and M = 26.60 (95% CI = 25.61 to 28.19) for the Mental Component Summary.

#### Self-Reported Physical Activity

The Simple Physical Activity Questionnaire (SIMPAQ), a questionnaire conducted as a personal interview consisting of five categories (time spent in bed, in sedentary behavior, walking, in structured exercise and in incidental physical activity), was used (SIMPAQ, 2020). It was developed for populations at high risk of increased levels of sedentary behavior, such as people suffering from psychiatric illness. The sum of all categories should equal 24 h, representing an average day in the previous week. There is no minimum time requirement set in any category. Intensities are not included except in the category of structured exercise, in which the number of sessions per week and time as well as intensity spent in each session is recorded. The intensity is elicited via a visual analog scale ranging from zero to ten. A large-scale validation study is being conducted to compare SIMPAQ results with accelerometer-based data (Rosenbaum and Ward, 2016). However, an exploratory study in a sample of healthy young adults was conducted. Significant correlations (p < 0.001) were found for moderate-to-vigorous physical activity between self-reported and accelerometer-based data (rho = 0.49). This study also includes the validation of the German language translation (Schilling et al., 2018).

#### Explicit Attitudes Toward Physical Activity

##### Physical Activity-Related Intention

The following one item was used to assess participants' intention to engage in physical activity: “How strong is your intention to be physically active in the next weeks and months?” Answer options range from 0 (no intention) to 5 (very strong intention). This measure is reliable and valid according to previous studies (Seelig and Fuchs, 2006; Gerber et al., 2011).

##### Physical Activity-Related Self-Concordance

Four subscales of motivation: intrinsic (“I would exercise because it is just fun”), identified (“I would exercise because I have good reasons to be physically active”), introjected (“I would exercise because otherwise I would have a guilty conscience”) and extrinsic (“I would exercise because others tell me to”) were assessed with the 12-item self-concordance scale (Seelig and Fuchs, 2006). All items are answered on a 6-point Likert scale ranging from 1 (not at all true) to 6 (completely true). The scores of the three questions pertaining to intrinsic motivation were added, and the mean derived to obtain a score for intrinsic motivation. The same was done for all subscales. This instrument has proven to be psychometrically sound (Seelig and Fuchs, 2006; Fuchs et al., 2012). In the present sample, the internal consistencies for all subscales was as follows: intrinsic: α = 0.71 (95% CI = 0.62–0.78), identified: α = 0.54 (95% CI = 0.40–0.65), introjected: α = 0.69 (95% CI = 0.60–0.77) and extrinsic: α = 0.72 (95% CI = 0.63–0.79). Cronbach's alpha for the overall scale was α = 0.69 (95% CI = 0.62–0.76). Despite the Cronbach's alpha for the subscale of identified motivation being below the acceptable threshold, this was accepted given the sample size and the acceptability of the overall scale value of Cronbach's alpha. The mean score across both groups was M = 3.59 (95% CI = 3.51–3.67).

##### Physical Activity-Related Self-Efficacy

Physical activity related self-efficacy was assessed with a 3-item score, with answers ranging from 0 (not at all confident) to 5 (100% confident in myself). The contents include self-efficacy beliefs regarding the initiation, maintenance and resumption of physical activity (e.g., “I feel confident to start with a new exercise activity”). The mean score is calculated from the three items, with higher scores representing higher self-efficacy levels. This questionnaire has been validated in a previous study (Fuchs, 2008). The internal consistency in the present sample was satisfactory (α = 0.82, 95% CI = 0.77–0.87). The mean score across both groups was M = 3.61 (95% CI = 3.45 to 3.76).

##### Physical Activity-Related Action Planning

A 5-item questionnaire was used to measure action planning (Sniehotta et al., 2005). Questions pertain to when, where, how, how often and with whom participants are usually physically active. Answers range from 1 (not at all true) to 4 (completely true). The mean represents the final score with higher values indicating higher levels of pre-planned physical activity. Reliability and validity have been established in previous studies (Sniehotta et al., 2005; Gerber et al., 2011). The internal consistency in the present sample was satisfactory (α = 0.81, 95% CI = 0.77–0.86). The mean score across both groups was M = 2.64 (95% CI = 2.54–2.75).

##### Physical Activity-Related Coping Planning

A 5-item questionnaire was used to evaluate coping planning (Sniehotta et al., 2005). On a 4-point Likert scale, participants give information pertaining to the extent to which they implement self-regulatory strategies to overcome barriers. One such question is “I have made a detailed plan regarding what to do in difficult situations in order to act in accordance to my intentions.” Answers range from 1 (not at all true) to 4 (completely true). The final score consists of the mean of all items. Psychometric properties have been evaluated resulting in acceptable reliability and validity of the scale (Sniehotta et al., 2005; Gerber et al., 2011). The internal consistency in the present sample was satisfactory (α = 0.84, 95% CI = 0.79–0.88). The mean score across both groups was M = 2.11 (95% CI = 1.99–2.22).

##### Physical Activity-Related Outcome Expectancies

Nine positive (e.g., “If I exercise or am physically active, I become more flexible”) and seven negative formulations (e.g., “If I exercise or am physically active, I could injure myself”) are provided to assess outcome expectancies (Fuchs, 1997). Answers range from 1 (not true) to 4 (completely true). The mean is calculated for positive as well as negative outcome expectancies, with higher scores indicative of higher positive or negative outcome expectancies correspondingly. This instrument has been proven to be reliable and valid (Fuchs, 1997; Fuchs et al., 2012). The internal consistency in the present sample was satisfactory for both positive (α = 0.77, 95% CI = 0.71–0.82) and negative outcome expectancies (α = 0.74, 95% CI = 0.67–0.80). The mean scores across both groups were M = 3.23 (95% CI = 3.16–3.30) for positive outcome expectancies and M = 2.00 (95% CI = 1.92–2.09) for negative outcome expectancies.

##### Physical Activity-Related Perceived Barriers

A 19-item tool was used to assess perceived barriers to physical activity (Krämer and Fuchs, 2010). Items take on the following nature: “I have too much work to do.” Answers range from 1 (almost never) to 4 (almost always). The mean is computed representing the overall score. Satisfactory psychometric properties have been proven in previous studies (Krämer and Fuchs, 2010; Kramer et al., 2014). The internal consistency in the present sample was satisfactory (α = 0.84, 95% CI = 0.80–0.88). The mean score across both groups was M = 2.13 (95% CI = 2.06–2.21).

##### Physical Activity-Related Social Support

A 7-item index, which has proven reliability and validity, was used to measure social support (Gerber et al., 2010). Questions addressed the extent to which the participant experiences support from their social network (e.g., “Close family or friends help me plan my exercise”). Answers range from 1 (almost never) to 4 (almost always). The overall score is represented by the mean with higher values indicating more social support compared to low values. The internal consistency in the present sample was satisfactory (α = 0.88, 95% CI = 0.84–0.90). The mean score across both groups was M = 2.43 (95% CI = 2.32–2.54).

#### Implicit Attitudes Toward Physical Activity

The computer-based Single Target-Implicit Association Test (ST-IAT) was used to assess implicit attitudes toward physical activity (Greenwald et al., 1998). It is a response-time based test using a target concept, in this case physical activity, and target categories, in this case good and bad. The visual stimuli were people exercising displaying no obvious affect as well as emoticons (smileys and frownies) (Greenwald et al., 2003). First, the participants were instructed to accurately categorize the stimuli by pressing a button corresponding to the respective target category. The test was initiated with a practice block containing 16 trials, in which only the emoticons had to be categorized as good (smileys) or bad (frownies). Following a fixation period of 250 ms, stimuli were presented until a response was collected. After the initial block, participants were instructed to assign emoticons and target images to one of the response categories, which presented sport either along with the good or bad category. The order of the categorization was counterbalanced across participants. Participants completed two blocks with 32 trials each, which were preceded by 16 practice trials to reduce learning effects. Upon incorrect response, a repetition took place at the end of the block. For statistical analysis, the D-score was calculated by dividing the ST-IAT raw scores (reaction time difference between the two block types) by the within-subject standard deviation of reaction times. The D-score can take on values between −2 and +2 and the interpretation goes as follows: 0.15 = slight, 0.35 = moderate, 0.64 = strong preference/aversion (Blanton et al., 2015). This version has been developed specifically for inactive in-patients suffering from depression using E-prime 2.0 (PST, USA) software and images obtained from Adobe Stock and has been pilot tested. General reliability and discriminant validity of the ST-IAT has been well-established (Greenwald et al., 2003; Bluemke and Friese, 2008; Blanton et al., 2015; Antoniewicz and Brand, 2016b).

Given that sustained attention may affect ST-IAT scores (Wright and Meade, 2012) this cognitive domain was assessed using a computerized Oddball Paradigm (Calitri et al., 2009) administered with E-Prime 2.0 (PST, USA). Task instructions were presented on the screen. Speed and accuracy were equally emphasized across both tests. The Oddball Paradigm required participants to press one button to frequent stimuli (75%) and another button to infrequent stimuli (25%). Visual stimuli were the letters “X” and “O” and the stimulus-response mapping was counterbalanced across participants. Following an inter-stimulus interval varying randomly between 800 and 1,500 ms, visual stimuli were presented over 250 ms and responses were allowed within 1,000 ms. The task encompassed a practice block of 10 trials and two test blocks with 40 trials each. Reaction time (on response-correct trials) accuracy were extracted for analyses. Reliability for the oddball paradigm has been proven in a previous study (Williams et al., 2005).

### Statistical Analyses

Descriptive statistics (M, SD, *n*, and %) were calculated for the total sample (*N* = 165) as well as both groups: pre-lockdown (*N* = 119) and post-lockdown (*N* = 46). Kolmogorov-Smirnov and Shapiro Wilk tests revealed that the assumptions of normality of the populations and homogeneity of population variance were violated in part. Hence, differences in potential confounders were measured with Brown Forsythe (BF) one-way Analyses of Variance (ANOVAs) to additionally adjust for unequal sample sizes. Differences in categorical variables were measured with χ^2^-tests. The BF procedure is robust, thus known to control Type 1 errors given heterogeneity of variance (Lix et al., 1996). To further analyze group differences in psychosocial health, physical activity and explicit as well as implicit attitudes toward physical activity, Analyses of Covariance (ANCOVAs) using age, sex and number of previous episodes as covariates as defined in the study protocol were conducted. Additionally, for implicit attitudes, sustained attention was controlled for by including reaction time on infrequent targets as a covariate. To test whether sex had a moderating effect, a two-factorial ANCOVA was carried out with the factors group (pre- vs. post), sex (female vs. male) and group by sex interactions. Effect sizes and partial eta-squared (η^2^) were computed to determine the relative degree of variance associated with each of the main effects. Statistical significance was set at *p* < 0.05 across all analyses. All analyses were conducted in SPSS software for Windows (version 26, IBM Corp., Armonk, NY, USA).

## Results

### Descriptive Statistics

As shown in Table 1, the sample consisted of approximately equal amounts of women and men who were on average middle-aged, single, employed before in-patient treatment and most of whom, were diagnosed with moderate recurrent depression. As reflected in BDI-scores at screening compared to baseline, depression scores reduced in line with being in in-patient treatment. HAMD scores differed statistically significantly between pre-lockdown (M = 14.03, SD = 5.10) and post-lockdown [M = 11.89, SD = 5.40, Brown Forsythe: *F*_(1, 77.8)_ = 5.39, *p* = 0.02, ANOVA: *F*_(1, 163)_ = 5.67, *p* = 0.02, η^2^ = 0.03], and were thus taken into account as co-variates in further analyses. Furthermore, the sample did not reach the weekly recommended 150 min of moderate-to-vigorous physical activity and perceived their fitness below average. Information about medication, specific antidepressants and secondary diagnoses is provided as supplementary material (see Supplementary Tables 1–3).

**Table 1:** Descriptive statistics and differences in potential confounders.

	**Total sample (*N* = 165) M (SD)**	**Pre-lockdown (*N* = 119) M (SD)**	**Post-lockdown (*N* = 46) M (SD)**	**Brown Forsythe**		**ANOVA**		
				***F***	***p***	***F***	***p***	***η^2^***
**Metric variables**								
Age (years)	41.88 (12.48)	41.94 (12.29)	41.74 (13.09)	0.01	0.93	0.01	0.93	<0.001
Education (years)	14.10 (3.41)	14.02 (3.58)	14.29 (2.94)	0.24	0.62	0.20	0.65	<0.001
BDI score at screening	28.72 (8.78)	28.14 (9.08)	30.32 (7.73)	2.01	0.16	1.72	0.19	0.01
BDI score at baseline	21.36 (9.83)	21.71 (9.95)	20.55 (9.62)	0.45	0.51	0.43	0.51	<0.001
HAMD at baseline	13.44 (5.25)	14.03 (5.09)	11.89 (5.39)	5.39	0.02	5.67	0.02	0.03
Prior episodes (number of)	2.90 (6.13)	3.24 (7.16)	2.13 (2.51)	1.95	0.16	1.05	0.31	0.01
Age (years) at 1st episode	32.03 (14.20)	31.77 (14.11)	32.62 (14.55)	0.11	0.74	0.11	0.74	<0.001
Pre-clinic PA (min/week)	33.29 (49.33)	28.85 (47.04)	41.55 (52.90)	1.70	0.20	1.82	0.18	0.01
Perceived fitness (1–10)	3.77 (1.56)	3.82 (1.56)	3.65 (1.56)	0.39	0.53	0.39	0.53	<0.001
	***n*** **(%)**	***n*** **(%)**	***n*** **(%)**	**χ^2^**	**phi**			
**Categorical variables**								
**Sex**				0.70	−0.06			
Women	84 (51)	63 (53)	21 (46)					
Men	81 (49)	56 (47)	25 (54)					
**Primary diagnosis**				2.96	0.13			
F32.1	39 (24)	30 (25)	9 (19)					
F32.2	22 (13)	18 (15)	4 (9)					
F32.3	2 (1)	1 (1)	1 (2)					
F33.1	71 (43)	50 (42)	21 (46)					
F33.2	31 (19)	20 (17)	11 (24)					
**Civil status**				2.82	−0.13			
Single	113 (68)	77 (65)	36 (78)					
Married	52 (32)	42 (35)	10 (22)					
**Employment (pre-clinic)**				0.03	−0.01			
Yes	124 (75)	89 (75)	35 (76)					
No	41 (25)	30 (25)	11 (24)					
**Yearly income**				1.18	0.09			
<50,000 CHF	55 (42)	38 (43)	17 (39)					
50,000–100,000 CHF	51 (38)	35 (40)	16 (36)					
>100,000 CHF	26 (20)	15 (17)	11 (25)					

*BDI, Beck Depression Inventory; HAMD, Hamilton Depression Score; PA, physical activity; Perceived fitness scale ranging from 1 = not fit to 10 = very fit. F32.1, major depressive disorder, single episode, moderate; F32.2, major depressive disorder, single episode, severe; F32.3, major depressive disorder, single episode, severe, with psychotic features; F33.1, major depressive disorder, recurrent, moderate; F33.2, major depressive disorder, recurrent, severe; CHF, Swiss Francs*.

### Between-Group Differences in Psychosocial Health and Stress

Table 2 shows that there were no statistically significant between-group differences with regard to psychosocial health (stress, sleep, physical and mental quality of life). Perceived stress scores in both groups were high (scores ranging from 0 to 40). The sample exhibited subthreshold insomnia (scores between 8 and 14). Both groups revealed relatively low scores in perceived physical and mental health with perceived mental health rated lower than physical health in both groups. Participants with a secondary diagnosis relating to physical health had lower scores for physical health (M = 46.93, SD = 8.58) than in the participants with no secondary diagnosis relating to physical health (M = 51.11, SD = 8.28).

**Table 2:** Between-group differences in psychosocial health.

	**Pre-lockdown**		**Post-lockdown**		**Brown Forsythe**		**ANCOVA**		
	**M**	**SD**	**M**	**SD**	***F***	***p***	***F***	***p***	***η* ^2^**
**Psychosocial health**									
Stress	36.88	5.84	37.55	4.57	0.43	0.51	3.76	0.05	0.03
Sleep	12.23	5.17	11.82	6.31	0.15	0.70	0.26	0.61	<0.001
PCS	48.95	8.75	49.87	8.45	0.36	0.55	0.01	0.91	<0.001
MCS	26.71	8.22	27.36	7.69	0.21	0.64	0.41	0.52	<0.001

*PCS, physical component scale of the SF-12; MCS, mental component scale of the SF-12*.

*Brown Forsythe without co-variates*.

### Between-Group Differences in Self-Reported Physical Activity and Explicit and Implicit Attitudes Toward Physical Activity

Table 3 shows that there were no statistically significant differences in self-reported physical activity levels. However, the post-lockdown group did achieve the recommended 150 min of moderate-to-vigorous physical activity per week (M = 170.10, SD = 149.74 min/week) whereas the pre-lockdown group did not (M = 137.42, SD = 144.70 min/week). Additionally, there were no statistically significant differences in explicit (intention, motivation, self-efficacy, planning, coping, positive and negative expectancies barrier and social support) or implicit attitudes toward physical activity. Results show that the sample had the intent to be physically active and they were most driven by intrinsic and identified motivation. Scores for planning physical activity, coping with physical activity related barriers, perceived barriers and social support were low in both groups (scores ranging from 1 to 6). Yet negative outcome expectancies were low and positive ones were high, indicating that the participants may have had more positive attitudes toward the expected outcomes they may gain from physical activity. These results, which may be interpreted as a leaning toward positive explicit attitudes, seem in accordance with the ST-IAT scores, which indicated slight implicit preference for physical activity in both groups.

**Table 3:** Between-group differences in self-reported physical activity and explicit and implicit attitudes toward physical activity.

	**Pre-lockdown**		**Post-lockdown**		**Brown forsythe**		**ANCOVA**		
	**M**	**SD**	**M**	**SD**	***F***	***p***	***F***	***p***	***η* ^2^**
**Self-reported physical activity**									
Minutes per week	137.42	144.70	170.10	149.74	2.05	0.16	0.82	0.37	0.01
**Explicit attitudes toward physical activity**									
Intention	3.82	1.05	3.88	1.03	0.34	0.56	0.09	0.76	<0.001
Intrinsic motivation	3.88	1.06	3.88	0.96	0.09	0.77	0.02	0.89	<0.001
Identified motivation	4.85	0.71	4.84	0.64	0.02	0.89	0.01	0.91	<0.001
Introjected motivation	3.57	1.05	3.53	0.92	<0.001	0.98	<0.001	1.00	<0.001
Extrinsic motivation	2.19	1.03	2.18	0.93	0.01	0.92	0.03	0.86	<0.001
Self-efficacy	3.62	0.99	3.52	1.01	0.53	0.47	1.67	0.20	0.01
Planning	2.62	0.68	2.74	0.61	1.51	0.22	0.32	0.57	<0.001
Coping	2.12	0.68	2.14	0.61	0.18	0.67	<0.001	0.95	<0.001
Positive expectancies	3.23	0.38	3.19	0.52	0.45	0.50	0.18	0.67	<0.001
Negative expectancies	2.06	0.57	1.86	0.46	5.62	0.02	2.69	0.10	0.02
Barriers	2.17	0.49	2.05	0.38	2.34	0.13	0.50	0.48	<0.001
Social support	2.33	0.71	2.58	0.72	2.88	0.09	2.96	0.09	0.02
**Implicit attitudes toward physical activity**									
D-Score	0.16	0.44	0.19	0.44	<0.001	0.94	0.42	0.52	<0.001

*Brown Forsythe without co-variates*.

Table 4 shows a small yet statistically significant difference between women and men [F_(1, 131)_ = 4.88, *p* = 0.03, η^2^ = 0.04), regarding positive outcome expectancies of physical activity with women reporting higher rates of positive outcome expectancies compared to men.

**Table 4:** Between-group differences in psychosocial health, self-reported physical activity and explicit/implicit attitudes toward physical activity, with group by sex interactions.

	**Group 1: Pre-lockdown** **(*****N*** **=** **119)**		**Group 2: Post-lockdown** **(*****N*** **=** **46)**		**Group**		**Sex**		**Group** **×** **sex**	
	**Women**	**Men**	**Women**	**Men**						
	**M (SD)**	**M (SD)**	**M (SD)**	**M (SD)**	***F***	***η^2^***	***F***	***η^2^***	***F***	***η^2^***
**Psychosocial health**										
Stress	37.89 (6.17)	35.87 (5.38)	37.35 (4.50)	37.71 (4.72)	3.47	0.03	0.06	<0.001	1.79	0.01
Sleep	12.90 (5.09)	11.48 (5.38)	11.65 (7.00)	11.96 (5.83)	0.22	<0.001	0.03	<0.001	0.48	<0.001
PCS	47.53 (9.56)	50.46 (7.23)	50.89 (8.08)	48.93 (8.85)	0.02	<0.001	0.16	<0.001	2.03	0.02
MCS	26.30 (8.94)	27.61 (7.81)	27.08 (7.40)	27.62 (8.10)	0.40	<0.001	0.12	<0.001	0.02	<0.001
**Self-reported physical activity**										
Minutes per week	130.63 (138.64)	144.16 (151.64)	161.20 (137.08)	177.58 (162.03)	0.80	0.01	0.21	<0.001	0.01	<0.001
**Explicit attitudes**										
Intention	3.84 (1.07)	3.80 (1.04)	3.63 (1.01)	4.08 (1.02)	0.16	<0.001	0.91	0.01	0.15	0.01
Intrinsic motivation	3.94 (1.05)	3.83 (1.08)	3.89 (0.97)	3.89 (0.97)	0.02	<0.001	0.30	<0.001	0.09	<0.001
Identified motivation	4.89 (0.74)	4.81 (0.70)	4.81 (0.81)	4.87 (0.46)	0.02	<0.001	0.03	<0.001	0.39	<0.001
Introjected motivation	3.49 (1.10)	3.66 (1.00)	3.62 (0.90)	3.46 (0.95)	<0.001	<0.001	<0.001	<0.001	1.08	0.01
Extrinsic motivation	2.14 (1.16)	2.24 (0.88)	1.94 (0.85)	2.38 (0.96)	0.02	<0.001	2.49	0.02	0.76	0.01
Self-efficacy	3.64 (0.96)	3.60 (1.03)	3.43 (1.02)	3.60 (1.01)	1.74	0.01	0.01	<0.001	0.45	<0.001
Implementation	2.72 (0.61)	2.52 (0.75)	2.74 (0.55)	2.75 (0.67)	0.26	<0.001	1.31	0.01	1.11	0.01
Coping	2.23 (0.65)	2.00 (0.70)	2.04 (0.70)	2.23 (0.52)	0.02	<0.001	0.38	<0.001	3.73	0.03
Positive expectancies	3.31 (0.37)	3.16 (0.37)	3.29 (0.61)	3.10 (0.42)	0.17	<0.001	4.88^*^	0.04	0.08	<0.001
Negative expectancies	2.00 (0.56)	2.12 (0.58)	1.77 (0.49)	1.93 (0.44)	2.72	0.02	3.87	0.03	0.10	<0.001
Barriers	2.16 (0.46)	2.18 (0.53)	2.08 (0.35)	2.03 (0.41)	0.47	<0.001	0.19	<0.001	0.14	<0.001
Social support	2.28 (0.72)	2.38 (0.70)	2.56 (0.68)	2.61 (0.77)	2.93	0.02	0.39	<0.001	0.02	<0.001
**Implicit attitudes**										
D-Score	0.13 (0.46)	0.19 (0.43)	0.20 (0.50)	0.15 (0.40)	0.24	<0.001	0.04	<0.001	0.92	0.01

* *p < 0.05*.

*PCS, physical component scale of the SF-12; MCS, mental component scale of the SF-12*.

## Discussion

The main finding from this analysis is that there are no statistically significant differences between in-patients with MDD recruited and assessed before and during/after the COVID-19-induced lockdown with regard to psychosocial health (stress, sleep, health-related quality of life), self-reported physical activity and explicit as well as implicit attitudes toward physical activity.

Hence, the COVID-19-induced lockdown is not likely to have impacted the baseline data collected during this ongoing trial. This is an important finding because the COVID-19 pandemic has had a negative impact on the conduct of clinical trials especially in vulnerable samples (van Dorn, 2020). The successful reuptake of recruitment and data collection in this trial was largely because the systems in place were linked to care as usual. Additionally, these activities took place on site with a maximum of two individuals in a room, wearing masks and practicing physical distancing. The continuation of quality research is needed especially in this time to overcome existing as well as COVID-19 related conditions. For example, research on physical activity behavior may be particularly important as it may have protective effects. It is known that regular moderate exercise contributes to a well-functioning immune system through reductions in inflammation, alterations in the composition of immune cells and relief of psychological stress (Simpson et al., 2015). The effects of physical activity on the immune system can be particularly meaningful with regard to viral diseases, hence, it is suggested that physical activity could be an important complement to preparing the immune system to fight COVID-19 infections (da Silveira et al., 2020). Additionally, being physically active has been shown to be associated with a lower prevalence of COVID-19 related hospitalizations (de Souza et al., 2020). When regarding the effect of physical activity on mortality caused by respiratory diseases in general, such as influenza, a study found that those adults performing low, moderate or frequent exercise were less at risk of mortality compared to those never or rarely engaging in exercise (Wong et al., 2008). Furthermore, a study conducted in elderly men examining different training patterns in relation to vaccine response found that both moderate and intense training patterns are associated with high antibody responses, which also last longer (de Araújo et al., 2015). In addition, regular physical activity during the pandemic may lead to fewer self-reported symptoms of depression and anxiety. This is especially true when comparing physical activity to no physical activity, or activity performed with lower volume and frequency. According to a rapid systematic review, there is no consensus regarding the exact volume and frequency of physical activity, however, in the included studies vigorous physical activity done regularly as opposed to irregularly was significantly associated with fewer depression symptoms (Wolf et al., 2020). This is of particular relevance for this sample population, which shows low levels of self-reported physical activity and may be at risk of decreased immune response.

One characteristic of the current pandemic contributing to psychological stress is social isolation, which has resulted from protective measures put in place (Clemente-Suárez et al., 2020). Isolation and the accompanying loneliness are associated with depression (Matthews et al., 2016) while depression is also associated with a small social network (Domènech-Abella et al., 2017). In combination this delineates the distinction between emotional and social loneliness and that both are present in symptoms of depression (Domènech-Abella et al., 2017). Hence, a hypothesis could be that people already suffering from depression may not notice the decline in social contact brought on by COVID-19 as much compared to healthy populations. Additionally, in the current sample, the participants were not in their everyday environment, but in in-patient treatment where social interactions are provided and encouraged (Holsboer-Trachsler et al., 2016). Another noteworthy point regarding psychosocial stress levels is that the current population had high levels of perceived stress in both groups. Hence, it could be hypothesized that the stress levels in the current sample of in-patients with MDD were at a level at which the addition of the pandemic may not have had a further impact. With regard to the lack of difference in sleep patterns, complaints may have been addressed by the structured nature of in-patient treatment and medication regimes. On the topic of health-related quality of life, it is known that people reporting increased symptoms of depression also generally report poorer health-related quality of life (lower scores in the chosen measuring instrument) involving physical, psychological and social domains which in turn may negatively affect functioning. The implications thereof are that dysfunctions can be targeted in tailored therapy settings (Daly et al., 2010). The association between poor health-related quality of life and increased depression symptoms has also been confirmed in people with major depression (Sivertsen et al., 2015). A lack of difference in psychosocial health may be explained by the population at hand. Previous evidence shows that there are negative influences of COVID-19 on psychosocial health, however particularly in children, older people and their care-givers. When considering people with psychiatric conditions it is especially those suffering from obsessive-compulsive disorder (OCD) and those at risk of relapse or discontinuation of therapy who are at risk (Dubey et al., 2020). In this population all participants were in in-patient care and receiving therapy, thus the possibility of relapse was very low. When looking at other research being done in the area of psychosocial distress in the context of COVID-19, studies can be found on university students (Villani et al., 2021), health care workers (Shayganfard et al., 2021) and the general population (Wang et al., 2020). Further indicating that a change in people already diagnosed with major depression has not been investigated and may, for the afore-mentioned reasons, not be particularly likely.

A lack of difference in physical activity in the context of COVID-19 has also been found in Swiss office workers. According to IPAQ assessments, 75% of the participants reached physical activity recommendations and there was no significant evidence of decreased physical activity comparing measures in January to April 2020. Seventeen per cent reported less activity, while 29% even reported more activity (Aegerter et al., 2021). Similar results are found in Iranian adults engaging in team sports. Here physical activity intensity did decrease, however, frequency increased and physical activity patterns were unrelated to mood (Aghababa et al., 2021). Increases in physical activity from screening to baseline in this study may be attributable to different patterns of behavior when living at home vs. being in in-patient treatment. The large standard deviations may be explained by the range of physical activity (0–150 min per week) permitted according to the inclusion criteria. Corresponding to the level of physical activity, the related explicit attitudes elicited from the questionnaires indicate a positive inclination. This can be explained by the fact that they all enrolled in a trial to facilitate a more physically active lifestyle. Hence, a certain interest in becoming more active could be expected as can also be confirmed when taking into consideration that intrinsic motivation levels were high, indicating that physical activity goals were concordant with other interests and values and less induced by external forces (Fuchs, 2008). Arguably, these results could be influenced by social desirability, which is a weakness of the assessment of explicit attitudes (Axt, 2017) and a selection bias occurring during the recruitment process. However, there is a positive tendency in explicit as well as implicit attitudes. Concordant explicit and implicit attitudes, especially with regard to physical activity, have proven to be advantageous for performing the behavior in future (Muschalik et al., 2019). Attitudes generally do not change quickly, building motivational self-regulation takes time, and deep seated automatic evaluations change slowly (Deutsch and Strack, 2006). Hence, no group differences in attitudes is in line with the current state of knowledge. It could be hypothesized that if attitudes toward physical activity actually would differ, they might differ both in a positive and negative direction. The COVID-19-induced lockdown has led to more leisure time and thus people may spend more of it being physically active (Cheval et al., 2020). Additionally, the protective benefits of physical activity may become even more tangible. On the other hand, anxiety regarding safe locations and types of physical activity during COVID-19 may contribute to more negative attitudes.

The relevance of the present study for research is that the ongoing trial can be continued with the assumption that baseline data may not have been contaminated. In addition, it may provide trials with similar designs, contents and population the basis for checking data quality and integrity and supporting findings. Furthermore, it is of clinical relevance to note the levels of self-reported physical activity. Thus, in-patient treatment may have the potential to be the platform from which to influence health behaviors such as physical activity. Along these lines, in-patients may be given the possibility to engage in physical activity and receive encouraged and positive feedback in continuing and increasing physical activity during in-patient care irrespective of when they were admitted.

The strengths of the current analysis are that potential COVID-19-induced interference with baseline data in this study have been monitored, thus contributing to data integrity. Meaningful co-variables for the variables of interest were collected and included in the analyses, hence, allowing for an optimized explanation of variance. The appropriate instruments were used for the measures, especially noteworthy is the population-specific ST-IAT. To investigate the accuracy of measures of depression severity, the HAMD score was checked for the influence of different researchers who performed the interviews and no differences were found. Additionally, the HAMD and BDI scores correlated positively, thus the assumption may be safe that the measuring method was accurate. The decrease in BDI score from screening to baseline is most likely because of the onset of treatment upon clinic admission.

Despite these strengths, there are limitations to be considered. This is a small sample of Swiss in-patients who were assessed at varying duration of stay and receiving a variety of treatments. Hence, the results cannot be generalized to all people with depression or indeed any other population. Given the cross-sectional nature of the data, the effect of the COVID-19-induced lockdown on psychosocial health and physical activity in people with major depression remains unknown and no causation can be inferred. There is a potential for selection bias, which may be visible in the group differences in HAMD scores. With this in mind, the possibility of systemic differences between the two groups does exist. Furthermore, the lack of difference between the two groups may also be explained by the smaller sample recruited and measured after the COVID-19-induced lockdown, which may have lead to decreased statistical power. Additionally, to investigate whether psychosocial stress, physical activity and attitudes toward physical activity in people suffering from MDD change over time in relation to the COVID-19 pandemic, longitudinal data are needed.

## Conclusion

In the present study, it can be said that the COVID-19-induced lockdown did not lead to differences in the current groups recruited and assessed before and during/after the lockdown. However, to assess changes in people with MDD with regard to psychosocial health, physical activity and attitudes toward physical activity longitudinal data are needed. For the ongoing trial, this means that baseline data gathered in in-patients suffering from depression who are physically inactive upon admission to in-patient treatment seem not to be impacted by the COVID-19-induced lockdown in March 2020 in Switzerland. For the future, an analysis of longitudinal data would be of importance in light of changes in psychosocial health and physical activity behavior brought on by COVID-19 discovered in healthy populations given that these aspects are also of particular importance for people with MDD. For other clinical trials continuing during this pandemic, it will be necessary to assess potential effects of COVID-19 on data and intervention delivery.

## Data Availability Statement

The raw data supporting the conclusions of this article will be made available by the authors, without undue reservation.

## Ethics Statement

The studies involving human participants were reviewed and approved by Ethikkommission Nordwest- und Zentralschweiz and Ethikkommission Bern. The patients/participants provided their written informed consent to participate in this study.

## Author Contributions

MG, JB, LD, AE, CI, MH, EH-T, UL, SL, TM, AO, NS, LZ, and OF have contributed to the design of the study. MG serves as principal investigator of the study. UL and NS (Basel), JB, AO, and AR (Riehen), CI and SM (Münchenbuchsee), and MH and TM (Solothurn) are responsible for the coordination and the recruitment of patients of the study in the four partner clinics. SL is responsible for the selection, programming and processing of data of the computer-based tests. EH-T, LZ, OF, and SB served as project advisors. RC and J-NK implement the recruitment and data assessment at the four partner clinics and were also responsible for the data entry, cleaning, and processing. RC and MG are responsible for the data analysis strategy applied in this paper. RC performed all statistical analyses and wrote the first draft of the manuscript. All authors contributed to manuscript revision, read, and approved the submitted version.

## Funding

The ongoing RCT was funded by the Swiss National Science Foundation (Grant No. 321003B-179353).

## Conflict of Interest

The authors declare that the research was conducted in the absence of any commercial or financial relationships that could be construed as a potential conflict of interest.

## Publisher's Note

All claims expressed in this article are solely those of the authors and do not necessarily represent those of their affiliated organizations, or those of the publisher, the editors and the reviewers. Any product that may be evaluated in this article, or claim that may be made by its manufacturer, is not guaranteed or endorsed by the publisher.
