# Supplementary material for: Psychosocial Health and Physical Activity in People With Major Depression in the Context of COVID-19
Source: Front Sports Act Living. 2021 Oct 29;3:685117. doi: 10.3389/fspor.2021.685117 (PMC8586655; doi:10.3389/fspor.2021.685117)
Supplement: Supplementary file 1 [file Table_1.DOCX]

**Supplementary material**

**Table S1.** Medication categories

|  | Total sample  (N = 165)  n (%) | Group 1  (N = 119)  n (%) | Group 2  (N=46)  n (%) |
| --- | --- | --- | --- |
| **Psychotropic medication** |  |  |  |
| Antidepressants (1) | 77 (47) | 55 (46) | 22 (48) |
| Antidepressants (>1) | 64 (39) | 47 (40) | 17 (37) |
| Psychostimulants (≥1) | 13 (8) | 10 (8) | 3 (6) |
| Anxiolytics (≥1) | 17 (11) | 10 (8) | 7 (15) |
| Sedatives (1) | 30 (18) | 20 (17) | 10 (22) |
| Antipsychotics (≥1) | 44 (27) | 30 (25) | 14 (30) |
| Antiepileptics (≥1) | 14 (9) | 11 (9) | 3 (7) |
| **Other medication** |  |  |  |
| Antihypertensives (≥1) | 31 (20) | 25 (21) | 6 (13) |
| Vitamins and minerals (≥1) | 48 (29) | 32 (27) | 16 (35) |
| Other (≥1) | 56 (34) | 35 (30) | 21 (46) |

**Table S2**. Antidepressants

|  | Total  (N = 165)  n (%) | Group 1  (N = 119)  n (%) | Group 2  (N = 46)  n (5) |
| --- | --- | --- | --- |
| **SARI** |  |  |  |
| Vortiotexin (5, 10, 15, 20 mg) | 33 (20) | 25 (21) | 8 (17) |
| **SNDRI** |  |  |  |
| Bupropion (50, 150, 300 mg) | 16 (10) | 8 (7) | 8 (17) |
| **SNRI** |  |  |  |
| Trittico (10, 50, 75, 100, 150, 200, 250, 300 mg) | 42 (25) | 31 (26) | 11 (24) |
| Venlafaxin (37.5, 75, 150, 187.5, 225, 300 mg) | 15 (9) | 8 (7) | 7 (15) |
| **SSNRI** |  |  |  |
| Duloxetin (15, 30, 60, 90, 120 mg) | 23 (14) | 18 (15) | 5 (11) |
| **SSRI** |  |  |  |
| Escitalopram (0.5, 5, 10, 20, 30, 40, 50 mg ) | 28 (17) | 19 (16) | 9 (19) |
| Citalopram (20, 30 mg) | 4 (2) | 3 (2) | 1 (2) |
| Fluoxetin (20, 40, 60, 80 mg) | 5 (3) | 4 (3) | 1 (2) |
| Paroxetin (20 mg) | 2 (1) | 2 (2) | 0 (0) |
| Sertralin (50, 100, 150, 200 mg) | 8 (5) | 7 (6) | 1 (2) |
| **Atypical** |  |  |  |
| Mirtazapin (15, 30, 45, 60 mg) | 16 (10) | 15 (13) | 1 (2) |
| **Tricyclic** |  |  |  |
| Anafranil (100, 150 mg) | 2 (1) | 1 (1) | 1 (2) |
| Saroten (50, 125 mg) | 2 (1) | 0 (0) | 2 (4) |
| Trimipramin (25, 50 mg) | 5 (3) | 3 (2) | 2 (4) |
| **Lithium** |  |  |  |
| Lithiofor, Quilonorm (12.2, 18.3, 25 mmol) | 9 (5) | 8 (7) | 1 (2) |
| **Herbal** | 2 (1) | 2 (2) | 0 (0) |

SARI = serotonin antagonist reuptake inhibitor. SNDRI = serotonin norepinepherine-dopamine, reuptake inhibitor. SNRI = serotonin-norepinepherine reuptake inhibitor. SSNRI = selective serotonin-norepinepherine reuptake inhibitor. SSRI = selective serotonin reuptake inhibitor.

**Table S3**. Secondary diagnoses

|  | Total  (N = 165)  n (%) | Group 1  (N = 119)  n (%) | Group 2  (N = 46)  n (%) |
| --- | --- | --- | --- |
| **Mental, behavioral and neurodevelopmental disorders** | | | |
| F10-F19 | 32 (19) | 24 (20) | 8 (17) |
| F40-F48 | 47 (28) | 39 (33) | 8 (17) |
| F50-F59 | 8 (5) | 6 (5) | 2 (4) |
| F60-F69 | 19 (11) | 15 (13) | 4 (9) |
| F70-F79 | 1 (1) | 1 (1) | 0 (0) |
| F80-F89 | 4 (2) | 3 (2) | 1 (2) |
| F90-F98 | 18 (11) | 13 (11) | 5 (11) |
| **Factors influencing health status and contact with health services** | | | |
| Z50 | 4 (2) | 4 (3) | 0 (0) |
| Z60 | 7 (4) | 5 (4) | 2 (4) |
| Z70 | 19 (11) | 15 (13) | 4 (9) |
| **Other** | 72 (44) | 50 (42) | 22 (48) |

F10-F19: mental and behavioral disorders due to psychoactive substance use. F40-48: Anxiety, dissociative, stress-related, somatoform and other nonpsychotic mental disorders. F50-59: behavioral syndromes associated with physiological disturbances and physical factors. F60-F69: disorders of adult personality and behavior. F70-79: intellectual disabilities. F80-89: Pervasive and specific developmental disorders. F90-98: behavioral and emotional disorders with onset usually occurring in childhood and adolescence. Z50: problems related to education, literacy, employment, housing, economic circumstances. Z60: problems related to social environment, upbringing, primary support group including family and psychosocial circumstances. Z70: problems related to medical facilities and other health care.
